# Platinum‐combination chemotherapy with or without immune‐checkpoint inhibitor in patients with postoperative recurrent non‐small cell lung cancer previously treated with adjuvant platinum‐doublet chemotherapy: A multicenter retrospective study

**DOI:** 10.1111/1759-7714.14992

**Published:** 2023-06-08

**Authors:** Kakeru Hisakane, Takehiro Tozuka, Satoshi Takahashi, Namiko Taniuchi, Nobuhiko Nishijima, Kenichiro Atsumi, Tetsuya Okano, Masahiro Seike, Takashi Hirose

**Affiliations:** ^1^ Department of Pulmonary Medicine and Medical Oncology Nippon Medical School Tamanagayama Hospital Tokyo Japan; ^2^ Department of Pulmonary Medicine and Oncology Graduate School of Medicine, Nippon Medical School Tokyo Japan; ^3^ Department of Respiratory Medicine Nippon Medical School Chiba Hokusoh Hospital Chiba Japan; ^4^ Department of Respiratory Medicine Nippon Medical School Musashikosugi Hospital Kanagawa Japan

**Keywords:** immune‐checkpoint inhibitor, non‐small cell lung cancer, platinum‐combination chemotherapy, postoperative recurrence

## Abstract

**Background:**

Rechallenge with platinum‐combination chemotherapy in patients with advanced non‐small cell lung cancer (NSCLC) after disease progression on platinum‐combination chemotherapy occasionally leads to a favorable response. The efficacy and safety of platinum‐combination chemotherapy with or without immune‐checkpoint inhibitor (ICI) for patients with recurrent NSCLC after surgery followed by adjuvant platinum‐doublet chemotherapy remains uncertain.

**Methods:**

Patients who relapsed after surgery plus adjuvant platinum‐doublet chemotherapy and received platinum‐combination chemotherapy with or without ICI between April 2011 and March 2021 at four Nippon Medical School hospitals were retrospectively analyzed.

**Results:**

Among 177 patients who received adjuvant platinum‐doublet chemotherapy after surgery, a total of 30 patients who received platinum‐combination rechemotherapy with or without ICI after relapse were included in this study. Seven patients received ICI‐combined chemotherapy. The median disease‐free survival (DFS) after surgery was 13.6 months. The objective response rate and disease‐control rate were 46.7% and 80.0%, respectively. The median progression‐free survival and overall survival were 10.2 and 37.5 months, respectively. Patients with longer DFS (≥12 months) had a better prognosis than others. The most common grade ≥3 toxicity associated with this treatment was neutropenia (33%). Grade ≥3 immune‐related adverse events were pneumonitis (14%) and colitis (14%). Treatment‐related deaths did not occur in this study.

**Conclusion:**

Platinum‐combination chemotherapy with or without ICI for patients with postoperative recurrent NSCLC who previously received adjuvant platinum‐doublet chemotherapy was effective and safe. In particular, this therapy may be promising for patients with longer DFS.

## Graphical abstract

Platinum‐combination chemotherapy with or without immune‐checkpoint inhibitors for patients with postoperative recurrent non‐small cell lung cancer (NSCLC) who previously received adjuvant platinum‐doublet chemotherapy was effective and safe. The outcome was noninferior to that of first‐line treatment for stage IV NSCLC.

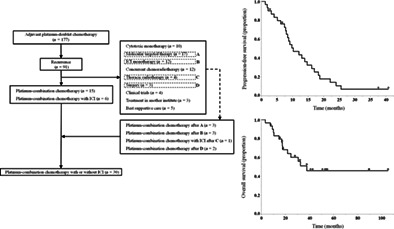

## INTRODUCTION

Platinum agents, such as cisplatin, carboplatin, and nedaplatin, are key anticancer drugs for the treatment of non‐small cell lung cancer (NSCLC). In recent decades, platinum agents in combination with other drugs (i.e., platinum‐combination chemotherapy) have been recommended as initial therapy for advanced NSCLC. Following the emergence of immune‐checkpoint inhibitors (ICIs), ICI monotherapy or ICI plus platinum‐combination chemotherapy has been established as the standard of care for the first‐line treatment of advanced NSCLC without driver mutations.^1^, ^2^, ^3^, ^4^, ^5^, ^6^ In the second‐ and later‐line settings, docetaxel with or without ramucirumab therapy,^7^, ^8^ pemetrexed monotherapy,^9^ and S‐1 monotherapy^10^ are recognized as the standard of care for patients with NSCLC after disease progression on platinum‐combination chemotherapy.

A meta‐analysis compared doublet chemotherapy with single‐agent treatment for advanced NSCLC which progressed after first‐line treatment (almost platinum‐combination chemotherapy). The results showed that doublet therapy was associated with significantly better objective response rate (ORR) and progression‐free survival (PFS) than single‐agent therapy. However, there was no significant difference in overall survival (OS) between the two arms, and a higher frequency of severe hematological toxicity was observed in patients who received doublet therapy.^11^ Randomized phase II trials comparing carboplatin plus pemetrexed with pemetrexed monotherapy for advanced NSCLC which progressed after platinum‐combination chemotherapy failed to demonstrate a benefit of the combination regimen on survival.^12^, ^13^ Moreover, a retrospective study compared rechallenge chemotherapy (almost platinum‐combination chemotherapy) with docetaxel alone for the second‐line treatment of patients with advanced NSCLC who responded to first‐line treatment. The ORR and OS were significantly better in the rechallenge group versus the docetaxel monotherapy group.^14^ Another retrospective study evaluated the efficacy of platinum‐doublet chemotherapy in patients with advanced NSCLC who experienced disease progression >6 months after the end of first‐line treatment. The analysis yielded favorable results (ORR: 30.4%; median PFS: 5.9 months; median OS: 12.5 months).^15^ Based on these findings, readministration of platinum‐combination chemotherapy may be promising for selected patients who achieved initial response to first‐line platinum‐combination chemotherapy.

Cisplatin‐based adjuvant chemotherapy is currently the standard of care for patients with completely resected stage II–IIIA NSCLC.^16^ Recently, adjuvant atezolizumab (anti‐programmed cell death‐ligand 1 [anti‐PD‐L1] blockade) improved disease‐free survival (DFS) compared with best supportive care after platinum‐combination chemotherapy for PD‐L1 positive resected stage II–IIIA NSCLC.^17^ Although the treatment strategy for recurrent NSCLC after surgery is controversial, systemic therapy (e.g., cytotoxic chemotherapy, molecular targeted therapy, ICIs, and a combination) is often adopted based on evidence for the care of stage IV NSCLC. Thus far, whether the efficacy and toxicity of platinum‐combination chemotherapy for patients with recurrent NSCLC after surgery who received adjuvant platinum‐combination chemotherapy is comparable to that of first‐line treatment for stage IV disease has not been thoroughly investigated.

Therefore, we conducted a multicenter retrospective study to investigate the efficacy and safety of platinum‐combination chemotherapy with or without ICI for postoperative recurrent NSCLC previously treated with adjuvant platinum‐doublet chemotherapy.

## METHODS

### Patient selection

Patients with NSCLC who relapsed after surgery followed by adjuvant platinum‐combination chemotherapy and received platinum‐combination chemotherapy with or without ICI between April 2011 and March 2021 at four Nippon Medical School hospitals were included in this study. Patients who underwent bevacizumab‐combined therapy, postoperative radiation therapy, molecular‐targeted therapy, ICI monotherapy, surgery, or radiotherapy as initial treatment after relapse were also eligible for enrollment. Notably, patients who received chemoradiotherapy as initial treatment after relapse were excluded. All clinical data were obtained from the medical records of patients. The pathological stage was determined using the eighth edition of the TNM classification developed by the International Union Against Cancer. The study was approved by the Ethics Committee Review Board at Nippon Medical School Hospital (Tokyo, Japan) (approval no. M‐2021‐021).

### Evaluation of efficacy and toxicity

The Response Evaluation Criteria in Solid Tumors version 1.1 were used to evaluate the best objective response through computed tomography. ORR was defined as the proportion of patients with complete response (CR) or partial response (PR). Disease‐control rate was defined as the proportion of patients with CR, PR, or stable disease. DFS was defined as the time from surgical resection until disease recurrence. PFS was defined as the time from the initiation of platinum‐combination chemotherapy with or without ICI until disease progression, death, or last follow‐up. OS was defined as the time from the initiation of platinum‐combination chemotherapy with or without ICI until death or last follow‐up. Toxicity was assessed according to the Common Terminology Criteria for Adverse Events version 5.0.

### Statistical analysis

Survival curves were produced based on the Kaplan–Meier method and compared using the log‐rank test. Univariate analyses for PFS or OS were performed using Cox proportional hazards regression analysis. All *p*‐values (two sided) <0.05 denoted statistically significant differences. The JMP statistical software package version 11 (SAS Institute) was employed for all data analyses.

## RESULTS

### Patient characteristics

**FIGURE 1:**
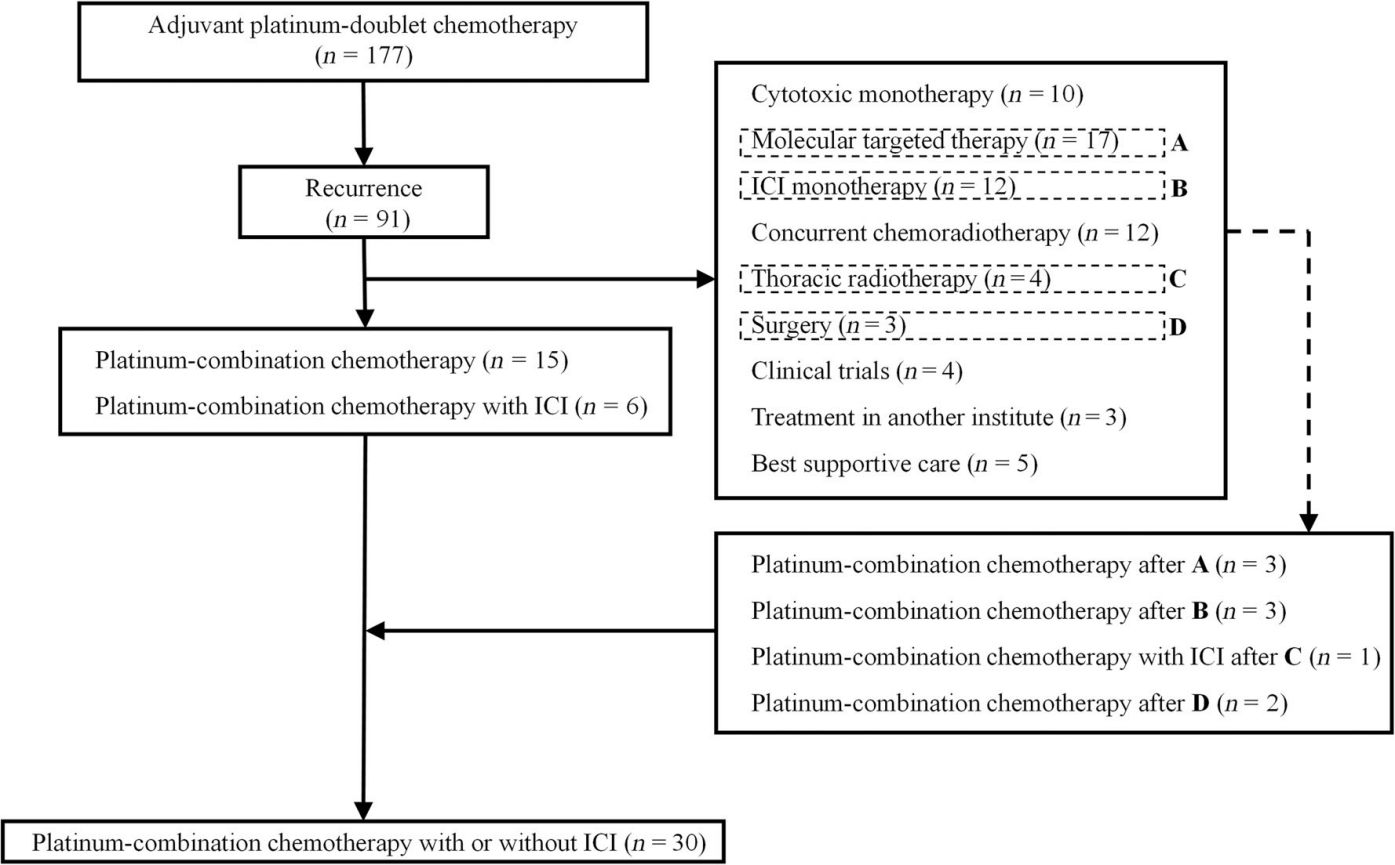
Patient inclusion flowchart. ICI, immune‐checkpoint inhibitor.

Figure 1 shows the patient flow diagram. A total of 177 patients received adjuvant platinum‐doublet chemotherapy after surgery. Of those, 91 patients experienced postoperative disease recurrence. A total of 21 patients received platinum‐combination chemotherapy with or without ICI as initial treatment after relapse. Moreover, three patients after molecular targeted therapy, three patients after ICI monotherapy, one patient after thoracic radiotherapy, and two patients after surgery at metastatic sites received platinum‐combination chemotherapy with or without ICI as later‐line therapy. Finally, 30 patients were eligible for inclusion in this study.

The characteristics of those 30 patients are summarized in Table 1. The median age was 67 years (range: 50–73 years), 67% were male, and 73% were smokers. All patients had an Eastern Cooperative Oncology Group performance status of 0 or 1. The most common histological classification was adenocarcinoma. Pathological stages II, III, IV were observed in four (13%), 23 (77%), and three patients (10%), respectively. Two of three patients with stage IV disease had pleural dissemination, while the remaining patient had a single brain metastasis. R0 and R1 resection was achieved in 27 (90%) and three (10%) patients, respectively. Cisplatin plus vinorelbine was the most commonly used regimen of adjuvant chemotherapy. Twenty‐five patients (83%) completed more than three cycles of adjuvant chemotherapy. Five patients (17%) discontinued adjuvant chemotherapy after one cycle due to patient preference (*n* = 3), adverse event (AE) (*n* = 1) (elevation of Krebs von den Lungen‐6 [KL‐6] serum levels without pneumonitis), and progressive disease (*n* = 1). Five patients (17%) received adjuvant thoracic radiotherapy. One patient (3%) underwent gamma knife radiosurgery for a single brain metastasis after primary resection. The median DFS was 13.6 months (range: 2.4–48.0 months).

**TABLE 1:** Patient characteristics.

Parameter	Value
Age, median (range), years	67 (50–73)
<65 years, *n*	12
≥65 years, *n*	18
Sex, *n*	
Male	20
Female	10
Smoking history, *n*	
Ever	22
Never	8
ECOG performance status, *n*	
0	12
1	18
Histology, *n*	
Adenocarcinoma	21
Squamous cell carcinoma	6
Other	3
PD‐L1 tumor proportion score, *n*	
≥50%	4
1–49%	4
<1%	8
Unknown	14
*EGFR* mutation, *n*	
Positive	3
Negative	24
Unknown	3
EML4‐ALK rearrangement, *n*	
Positive	2
Negative	20
Unknown	8
Pathological stage, *n*	
II	4
III	23
IV	3
Surgical procedure, *n*	
Lobectomy	29
Pneumonectomy	1
Adjuvant chemotherapy, *n*	
Cisplatin/vinorelbine	17
Cisplatin/S‐1	7
Carboplatin/S‐1	4
Carboplatin/pemetrexed	1
Cisplatin/etoposide	1
Adjuvant radiotherapy, *n*	
Thoracic radiotherapy	5
Gamma knife radiosurgery	1
None	24

Abbreviations: ECOG, Eastern Cooperative Oncology Group; EGFR, epidermal growth factor receptor; EML4‐ALK, echinoderm microtubule‐associated protein‐like 4‐anaplastic lymphoma kinase; PD‐L1, programmed cell death‐ligand 1.

### Response and survival

Various therapeutic regimens were used in this study (Table 2). Seven (23%) of 30 patients received ICI‐combined chemotherapy. Table 3 describes the therapeutic responses. CR, PR, stable disease, and progressive disease were observed in four, 10, 10, and four patients, respectively (two patients did not have evaluable lesions due to post‐resection of metastatic lesions). The ORR and disease‐control rate were 46.7% (95% confidence interval [CI]: 30.2%–63.9%) and 80.0% (95% CI: 62.7%–90.5%), respectively.

**TABLE 2:** List of platinum‐combination chemotherapy with or without immune‐checkpoint inhibitor.

Regimen	Number of patients
Cisplatin/pemetrexed/bevacizumab^a^	1
Carboplatin/pemetrexed/bevacizumab^b^	7
Cisplatin/pemetrexed^c^	3
Carboplatin/pemetrexed^d^	4
Carboplatin/nab‐paclitaxel/bevacizumab	1
Carboplatin/nab‐paclitaxel	2
Carboplatin/paclitaxel/bevacizumab	1
Carboplatin/docetaxel/bevacizumab	1
Cisplatin/docetaxel	1
Cisplatin/S‐1	1
Carboplatin/etoposide	1
Cisplatin/pemetrexed/pembrolizumab	1
Carboplatin/nab‐paclitaxel/pembrolizumab^e^	4
Carboplatin/nab‐paclitaxel/atezolizumab	1
Carboplatin/paclitaxel/bevacizumab/atezolizumab	1

Abbreviations: Nab, nanoparticle albumin‐bound.

^a^ This patient received cisplatin plus pemetrexed plus bevacizumab for the first two cycles, and carboplatin plus pemetrexed plus bevacizumab for the latter two cycles.

^b^ One of seven patients did not receive maintenance therapy with pemetrexed plus bevacizumab.

^c^ One of three patients did not receive maintenance therapy with pemetrexed.

^d^ Three of four patients did not receive maintenance therapy with pemetrexed.

^e^ One of four patients did not receive maintenance therapy with pembrolizumab.

**TABLE 3:** Response to platinum‐combination chemotherapy with or without immune‐checkpoint inhibitor.

Outcome	
Complete response, *n*	4
Partial response, *n*	10
Stable disease, *n*	10
Progressive disease, *n*	4
Not evaluated, *n*	2
Objective response rate, %	46.7
Disease control rate, %	80.0

**FIGURE 2:**
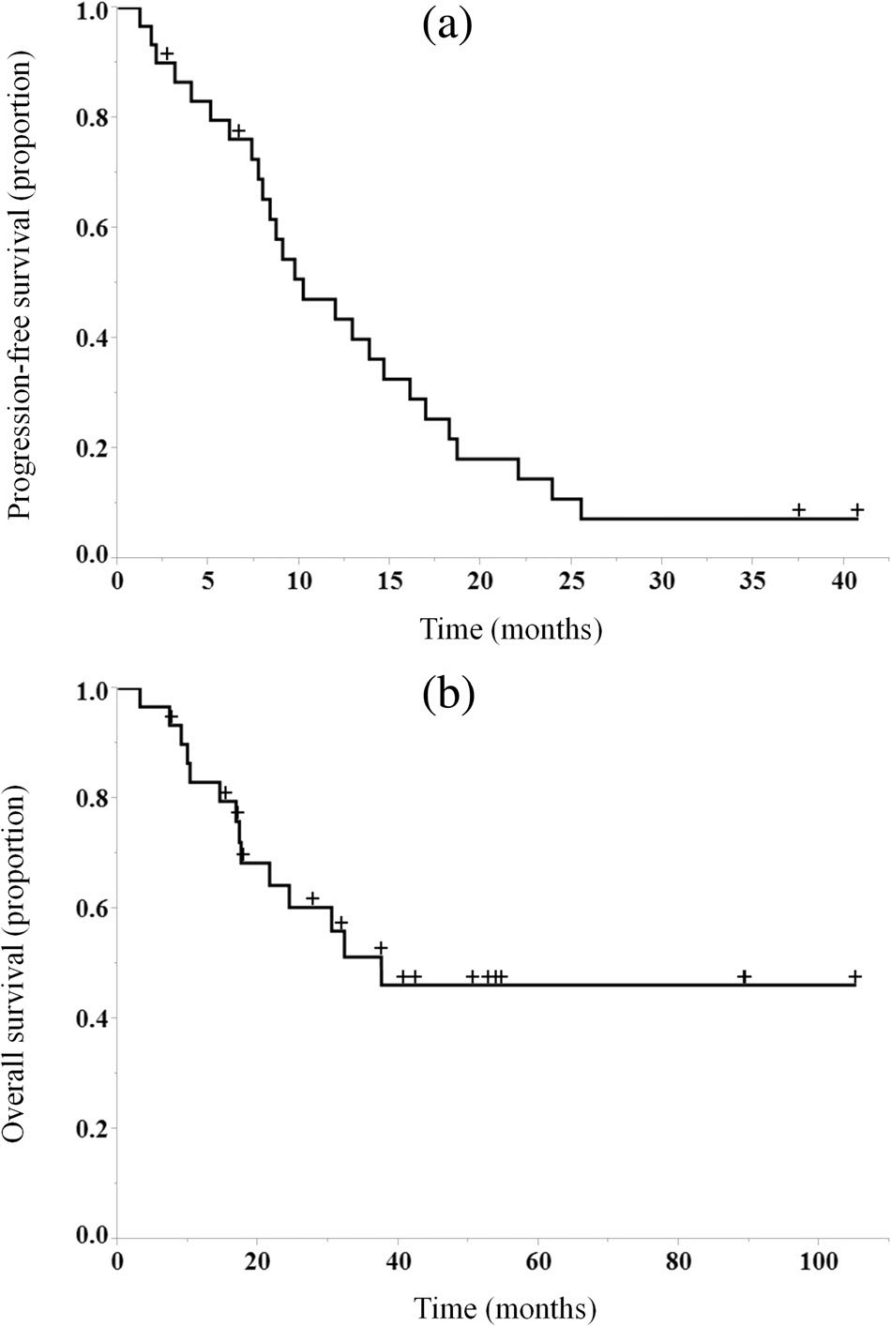
Kaplan–Meier curves for progression‐free survival (a) and overall survival (b) in all patients.

The median follow‐up time was 41.6 months (range: 7.7–105 months). The median PFS was 10.2 months (95% CI: 7.7–14.6 months) and the 1‐year PFS rate was 43.5% (95% CI: 26.7–61.9%) (Figure 2a). The median OS was 37.5 months (95% CI: 17.5 months–not reached), and the 3‐year OS rate was 51.3% (95% CI: 32.6–69.7%) (Figure 2b).

**FIGURE 3:**
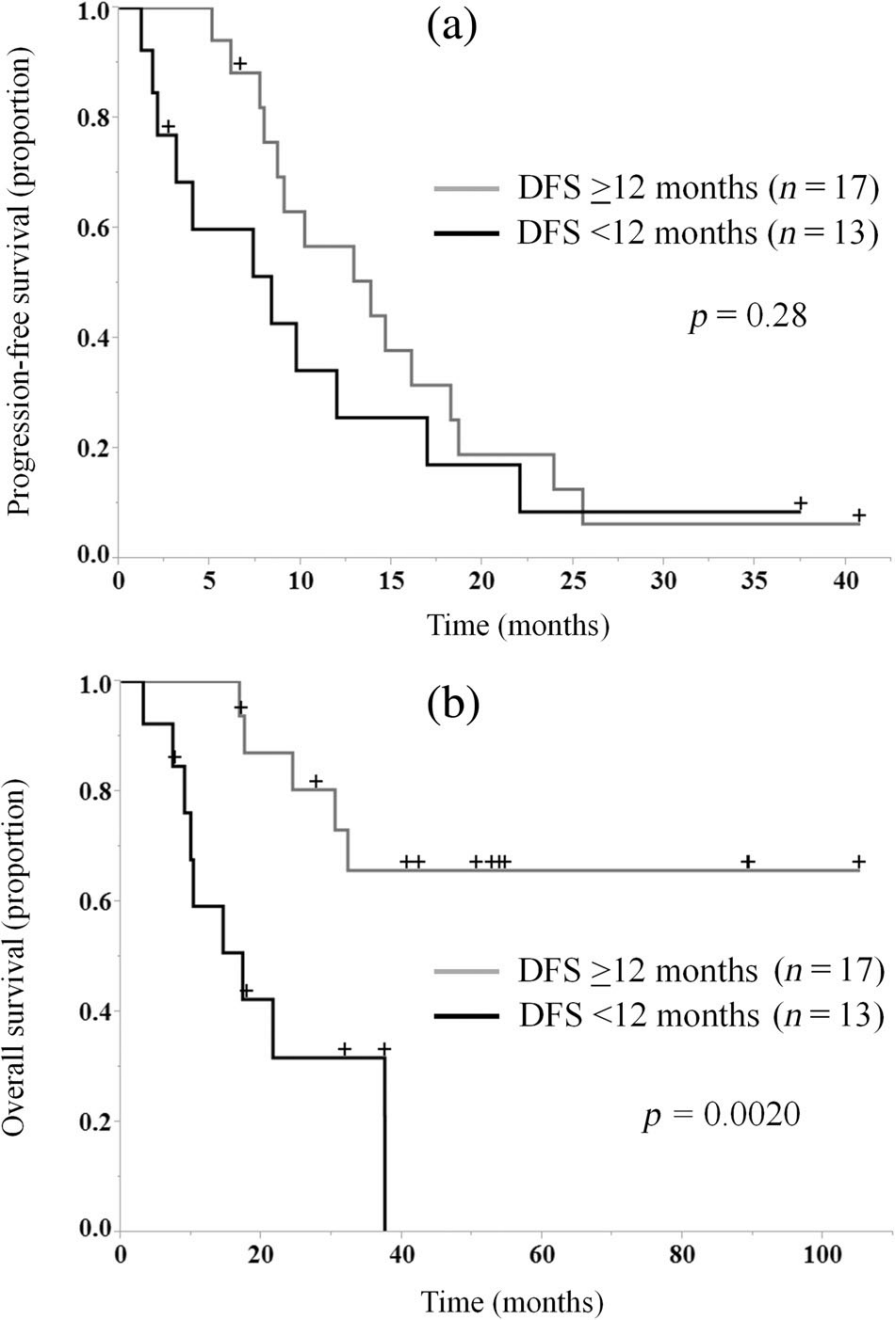
Kaplan–Meier curves for progression‐free survival (a) and overall survival (b) in all patients according to disease‐free survival (DFS).

Table 4 shows factors for PFS and OS identified through a univariate analysis. There was no factor predicting PFS, while DFS was identified as a predictor of OS. There was no statistically significant difference in PFS between patients with DFS <12 months and those with DFS ≥12 months (*p* = 0.28). In contrast, OS was significantly longer in patients with DFS ≥12 months versus those with DFS <12 months (*p* = 0.0020) (Figures 3a,b).

**TABLE 4:** Univariate analyses of factors for progression‐free survival and overall survival.

Parameters	*n*	Progression‐free survival		Overall survival	
		HR (95% CI)	*p*‐value	HR (95% CI)	*p*‐value
Age					
<65 years	12	1.02 (0.46–2.22)	0.96	0.44 (0.12–1.32)	0.15
≥65 years	18	–		–	
Sex					
Female	10	0.87 (0.37–1.93)	0.74	0.56 (0.59–6.49)	0.32
Male	20	–		–	
Smoking history					
Never	8	1.41 (0.54–3.28)	0.46	0.35 (0.05–1.30)	0.13
Ever	22	–		–	
Pathological stage					
II	4	0.79 (0.19–2.32)	0.70	0.42 (0.02–2.14)	0.35
III, IV	26	–		–	
Disease‐free survival					
<12 months	13	1.53 (0.68–3.32)	0.29	5.05 (1.69–16.9)	0.0038
≥12 months	17	–		–	
ICI combination					
No	23	1.54 (0.66–4.26)	0.33	0.91 (0.28–4.08)	0.89
Yes	7	–		–	

Abbreviations: CI, confidence interval; HR, hazard ratio; ICI, immune‐checkpoint inhibitor.

### Toxicity

Treatment‐associated toxicities are summarized in Table 5. Four patients (13%) discontinued treatment due to toxicity. Treatment‐related deaths did not occur in this study. Concerning nonimmune‐related adverse events (non‐irAEs), the most frequent grade ≥3 AE was neutropenia (33%). Febrile neutropenia was observed in two patients (7%). Grade ≥3 anemia (*n* = 3; 10%) and thrombocytopenia (*n* = 3; 10%) were also observed. Grade ≥3 non‐hematological AEs were lung infection (*n* = 1; 3%) and hyponatremia (*n* = 1; 3%). Among the seven patients treated with the ICI‐combined regimen, the following irAEs occurred: grade 4 pneumonitis in one patient (14%); grade 3 colitis in one patient (14%); and grade 2 pruritus in one patient (14%). Systemic corticosteroids were administered in two patients (pneumonitis and colitis).

**TABLE 5:** Adverse events.

Non‐irAE (≥grade 3)	Grade 3, *n*	Grade 4, *n*	Grade 3 or 4, *n* (%)
Neutropenia	8	2	10 (33)
Febrile neutropenia	2	0	2 (7)
Anemia	1	2	3 (10)
Thrombocytopenia	2	1	3 (10)
Lung infection	1	0	1 (3)
Hyponatremia	0	1	1 (3)

irAE (any)	Grade 2, *n*	Grade 3, *n*	Grade 4, *n*	Grade 3 or 4, *n* (%)
Pneumonitis	0	0	1	1 (14)
Colitis	0	1	0	1 (14)
Pruritus	1	0	0	0 (0)

Abbreviations: irAE, immune‐related adverse event.

## DISCUSSION

In this study, we revealed that platinum‐combination chemotherapy with or without ICI for patients with recurrent NSCLC after resection who previously received adjuvant platinum‐doublet chemotherapy was effective and safe.

According to the results of previous randomized phase III trials,^3^, ^4^, ^5^, ^6^ platinum‐combination chemotherapy and platinum‐combination chemotherapy plus ICI as first‐line treatment for metastatic NSCLC were associated with an ORR of 18.9%–48.0% and 47.6%–63.5%, median PFS of 4.8–6.8 months and 6.4–8.8 months, and median OS of 11.3–14.7 months and 15.9–19.2 months, respectively. Our results were encouraging, demonstrating an ORR of 46.7%, median PFS of 10.2 months, and median OS of 37.5 months, which were noninferior to those recorded in the aforementioned trials. Thus far, only a few retrospective studies have evaluated the efficacy of platinum‐combination chemotherapy in patients with relapsed NSCLC on postoperative adjuvant platinum‐doublet chemotherapy.^18^, ^19^ A summary of these studies, including the present study, is provided in Table 6. Our findings were somewhat superior to those observed in the previous two studies. This difference may be attributed to two reasons. First, in the present study, ICI‐combined chemotherapy was administered in seven of the 30 patients. Second, even among the 23 patients who received chemotherapy alone, seven cases were subsequently treated with ICIs in the later‐line setting. Conversely, ICIs were not administered in the previous two studies. In the univariate analysis, the addition of ICIs to platinum‐combination chemotherapy slightly prolonged PFS (not statistically significant); however, it did not exert an effect on OS (Table 4, Figure S1A,B). This may be explained by the fact that 30% of the patients (7/23) in the chemotherapy alone group received ICIs as post‐treatment, compared with 0% (0/7) in the ICI‐combined chemotherapy group. ICIs may contribute to survival regardless of the time of its administration. However, considering the slight effect on PFS, the use of ICIs plus chemotherapy may be a preferable option. Further study in a larger cohort is warranted to confirm this finding.

**TABLE 6:** Comparison with previous studies on platinum‐combination chemotherapy for patients with postoperative recurrent non‐small cell lung cancer previously treated with adjuvant platinum‐doublet chemotherapy.

Author, Reference no.	*n*	Regimen	DCR (%)	ORR (%)	Median PFS (months)	Median OS (months)
Imai et al.^18^	16	Platinum/pemetrexed/±bevacizumab (*n* = 11)	81.2	31.2	6.5	28.0
		Platinum/taxane/±bevacizumab (*n* = 3)				
		Other (*n* = 2)				
Valdes et al.^19^	25	Platinum/taxane^a^	66.7	29.2	Not shown	18.4
		Other				
Present study	30	Platinum/pemetrexed/±bevacizumab (*n* = 15)	80.0	46.7	10.2	37.5
		Platinum/taxane/±bevacizumab (*n* = 6)				
		Platinum/other (*n* = 2)				
		Platinum‐combination with ICI (*n* = 7)				

Abbreviations: DCR, disease control rate; ICI, immune‐checkpoint inhibitor; ORR, objective response rate; OS, overall survival; PFS, progression‐free survival.

^a^ The number of patients was not described.

We found that DFS ≥12 months was a favorable factor for OS. Patients with a longer DFS may have tumors that are responsive to postoperative platinum‐combination chemotherapy. For stage IV NSCLC, rechallenge with platinum‐combination chemotherapy is effective in patients who responded to first‐line platinum‐combination chemotherapy.^14^, ^15^ Our findings were consistent with previous results, suggesting that re‐challenge with platinum‐combination chemotherapy after postoperative recurrence in patients who achieved long response to adjuvant platinum‐doublet therapy may be promising. However, patients with a longer DFS may represent a favorable prognosis population regardless of treatment after recurrence. There was no statistically significant difference in PFS between patients with DFS <12 months and DFS ≥12 months. Nevertheless, patients with DFS ≥12 months showed a trend toward longer PFS (Figure 3a). In the chemotherapy alone group, the difference in PFS between these two groups was more pronounced (Figure S2). Addition of ICIs to platinum‐combination chemotherapy may be more beneficial for patients with DFS <12 months.

Regarding toxicity, platinum‐combination chemotherapy with or without ICIs for postoperative recurrent NSCLC previously treated with adjuvant platinum‐doublet chemotherapy was well tolerated. In previous trials comparing platinum‐combination chemotherapy with platinum‐combination chemotherapy plus ICIs for patients with advanced NSCLC, the rates of hematological toxicities, such as grade ≥3 neutropenia, grade ≥3 febrile neutropenia, grade ≥3 anemia, and grade ≥3 thrombocytopenia, were 11%–32%, 2%–9%, 5%–29%, and 4%–9%, respectively.^3^, ^4^, ^5^, ^6^ Our results were generally consistent with those previously reported. The nonhematological toxicities and irAEs associated with the present treatments were also manageable.

This study has four main limitations. First, this was a retrospective observational investigation. However, it is difficult to conduct prospective clinical trials because of the limited number of eligible patients. Second, the sample size of this study was small. This was due to the small number of institutions that participated in this study. Third, three patients with pathological stage IV disease were included in this study. Postoperative chemotherapy for these patients may not be strictly called “adjuvant chemotherapy”. Finally, the efficacy and safety of each regimen could not be adequately assessed because of the diversity of treatment regimens. In recent years, ICIs have become the mainstay of postoperative adjuvant treatment for NSCLC.^17^, ^20^ Hence, it is likely that the recruitment of eligible patients for such studies in the future will be challenging. Further well‐designed studies evaluating the efficacy and tolerability of platinum‐combination chemotherapy with or without ICIs for patients with recurrent NSCLC after surgery who previously received adjuvant ICIs are warranted.

In summary, we demonstrated that platinum‐combination chemotherapy with or without ICIs for patients with postoperative recurrent NSCLC who previously received adjuvant platinum‐doublet chemotherapy was effective and safe. This outcome is comparable to that of first‐line treatment for stage IV disease. In particular, this treatment option may be promising for patients who achieved long response to adjuvant platinum‐combination therapy.

## AUTHOR CONTRIBUTIONS

All authors had full access to the data in the study and take responsibility for the integrity of the data and accuracy of the data analysis. K.H., T.H.: Conceptualization; K.H., T.H.: Methodology; K.H., T.H., S.T., N.T., N.N.: Investigation; K.H.: Formal analysis; K.H., T.H., S.T., N.T., N.N., K.A., T.O.: Resources; K.H.: Writing – original draft; K.H., K.A., T.O., M.S., T.H.: Writing – review and editing; K.H.: Visualization; T.H.: Project administration; M.S., T.H.: Supervision; K.H.: Validation.

## CONFLICT OF INTEREST STATEMENT

M. Seike received funding from Taiho Pharmaceutical, Chugai Pharmaceutical, and MSD; and honoraria for lectures from Taiho Pharmaceutical, Chugai Pharmaceutical, Ono Pharmaceutical, Bristol‐Meyers Squibb, and MSD. T. Hirose received funding from Taiho Pharmaceutical, and Chugai Pharmaceutical; and honoraria for lectures from Taiho Pharmaceutical, Chugai Pharmaceutical, Bristol‐Meyers Squibb, and MSD. The other authors have no potential conflicts of interest.

## Supporting information


Figure S1.

Figure S2.
