# Supplementary figures and images for: Platinum‐combination chemotherapy with or without immune‐checkpoint inhibitor in patients with postoperative recurrent non‐small cell lung cancer previously treated with adjuvant platinum‐doublet chemotherapy: A multicenter retrospective study
Source: Thorac Cancer. 2023 Jun 8;14(21):2069–76. doi: 10.1111/1759-7714.14992 (PMC10363783; doi:10.1111/1759-7714.14992)

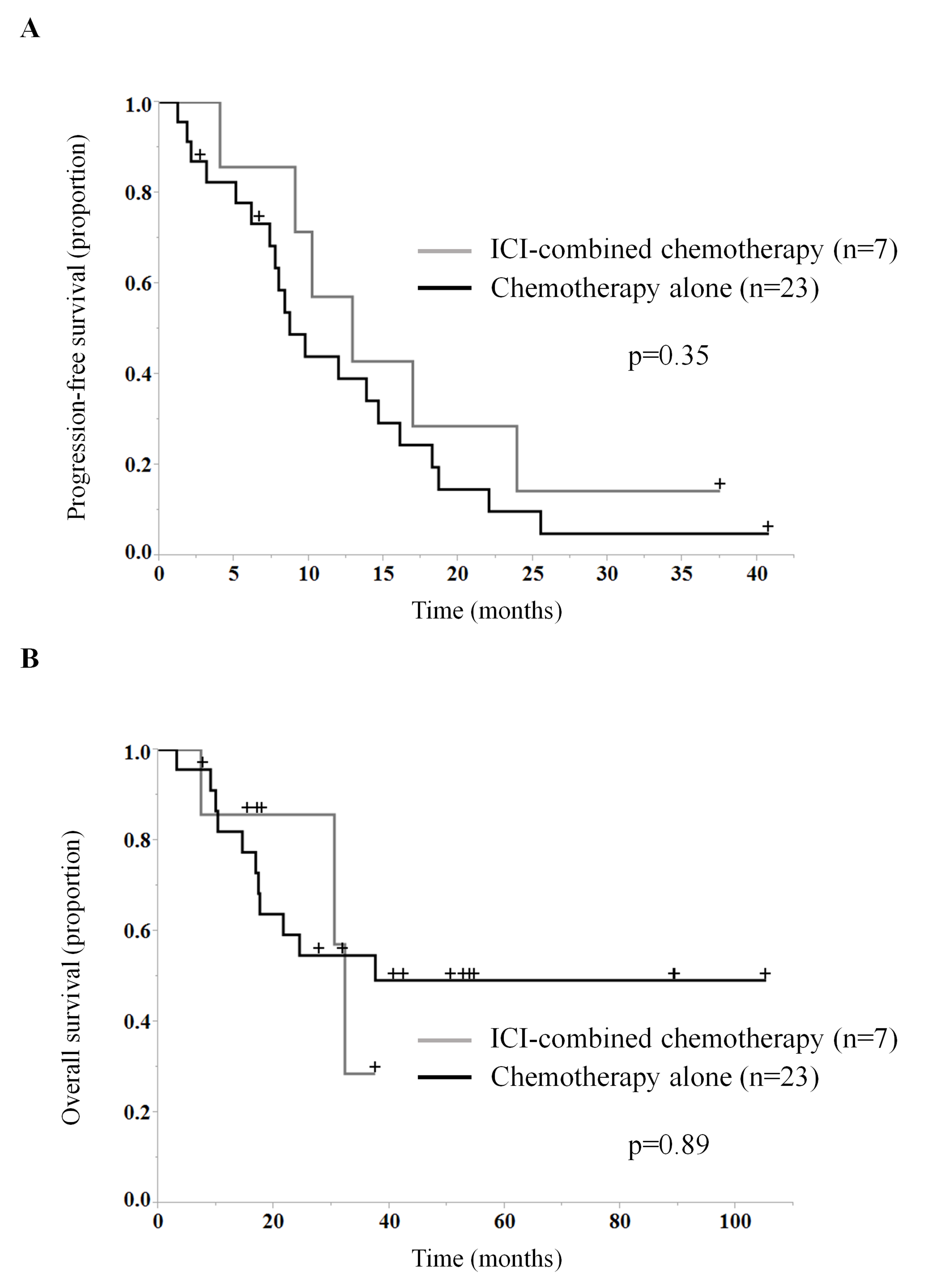

Supplement: Supplementary file 1 — Figure S1. [file TCA-14-2069-s002.tiff]

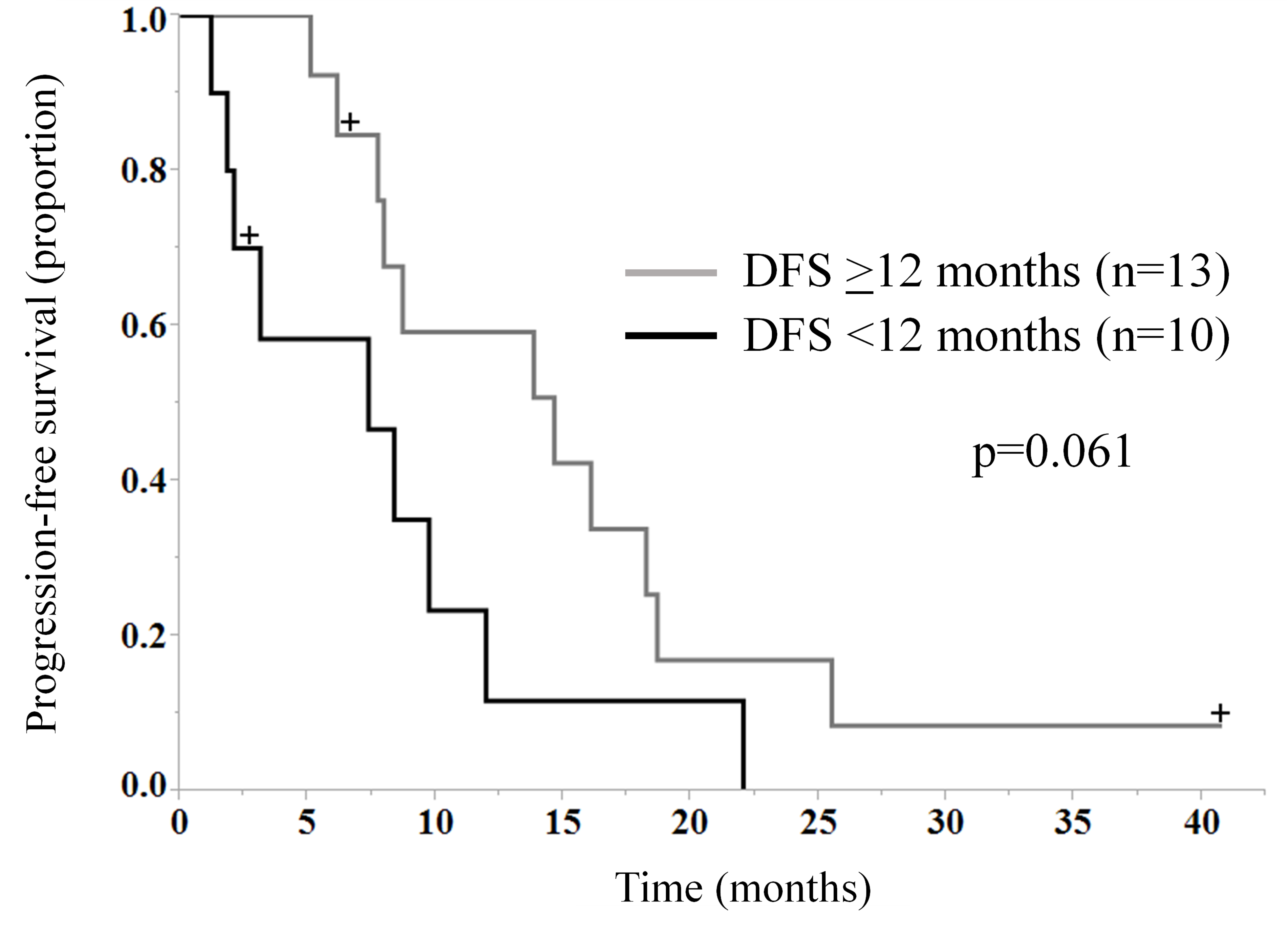

Supplement: Supplementary file 2 — Figure S2. [file TCA-14-2069-s001.tiff]
